# Elucidating the Therapeutic Mechanism of Danggui Liuhuang Decoction in Hyperthyroid Kidney Disease: An Integration of Network Pharmacology and Metabolomics

**DOI:** 10.1155/ije/5513418

**Published:** 2025-09-28

**Authors:** Yuxin Jiang, Zijian Wang, Yuechao Zhao, Qian Yu, Lili Weng, Chunping Xiao

**Affiliations:** School of Pharmacy, Changchun University of Chinese Medicine, Changchun, Jilin, China

**Keywords:** Danggui Liuhuang decoction, metabonomics, network pharmacology, pharmacodynamics

## Abstract

**Background and Objective:** Danggui Liuhuang decoction (DLD) demonstrates pharmacological efficacy in the treatment of hyperthyroid kidney disease (HKD). However, the underlying therapeutic mechanism remains inadequately understood. This study aims to elucidate the therapeutic mechanism of DLD in HKD rats by integrating the determination of effective component content, network pharmacology, in vivo verification, and metabolomics, with a focus on the alterations in metabolites and metabolic pathways.

**Methods:** The concentrations of 12 components, including berberine, in DLD were quantified using high-performance liquid chromatography. Network pharmacology was utilized to investigate the interactions between drug targets and disease targets and to predict functional pathways. A rat model of HKD was established to verify the therapeutic mechanism of DLD, which was further elucidated through metabolomics analysis.

**Results:** The quantification of 12 components, including berberine, in DLD was successfully achieved. Key targets such as TNF and AKT1, along with the PI3K/AKT and MAPK signaling pathways, were identified as critical pathways through network pharmacology analysis. Animal experiments robustly demonstrated the therapeutic efficacy of DLD on HKD, corroborating the network pharmacology findings via histopathological analysis, ELISA, and Western blot (WB). Metabolomics studies revealed significant alterations in 49 metabolites pre- and posttreatment, with notable changes in linoleic acid metabolism and arachidonic acid metabolism pathways. Linoleic acid, arachidonic acid, and 5-HETE were identified as potential biomarkers.

**Conclusion:** The therapeutic efficacy of DLD in HKD rats was substantiated, and the underlying mechanisms were preliminarily elucidated, thereby providing a foundation for further investigation into the pharmacodynamic substances and mechanisms of action.

## 1. Introduction

Hyperthyroidism is characterized by the excessive production of thyroid hormones, resulting in symptoms such as nervousness, sweating, heat intolerance, palpitations, increased appetite, and weight loss [1], as well as various complications, including kidney injury [2]. The current therapeutic strategies for managing hyperthyroidism and its associated complications predominantly involve the administration of antithyroid medications or the surgical intervention of thyroidectomy [3]. However, approximately 13% of patients undergoing treatment with antithyroid drugs report adverse allergic or toxic reactions [4]. Traditional Chinese medicine (TCM) presents distinct advantages in the management of endocrine disorders, owing to its holistic approach and a lower incidence of side effects [5].

Danggui Liuhuang decoction (DLD) is a traditional Chinese herbal formulation with a long-standing history of use, originating from the esteemed medical text “Lanshi Micang,” authored by Dongyuan Li during the Jin Dynasty. This formulation comprises Angelicae Sinensis Radix, Rehmanniae Radix, Rehmanniae Radix Praeparata, Scutellariae Radix, Coptidis Rhizoma, Phellodendri Chinensis Cortex, and Astragali Radix. Recognized for its therapeutic efficacy, DLD has been included in the first batch of the Catalogue of Famous Ancient Prescriptions. Traditionally, it has been employed to treat night sweats, which, according to the theoretical framework of TCM, are attributed to yin deficiency leading to hyperactivity of fire. In contemporary clinical practice, DLD has extensive applications and is frequently utilized in the management of conditions such as hyperthyroidism, diverse forms of hyperhidrosis [6], and complications associated with diabetes mellitus. The clinical outcomes associated with its use are notably significant.

Nonetheless, the intricate composition of TCM compounds presents significant challenges in elucidating their pharmacological mechanisms [7]. Network pharmacology offers a promising approach to uncovering the potential mechanisms of drug interventions in diseases by integrating biological system network analysis [8]. This methodology demonstrates considerable potential in investigating the pharmacological mechanisms of TCM compounds. By employing network pharmacology, researchers can analyze the complex interactions between drugs and diseases, predict drug targets and possible biological pathways, and establish a foundation for subsequent in vitro and in vivo verification [9]. Metabolomics technology offers substantial advantages in elucidating alterations in body composition, rendering it an invaluable tool for investigating the mechanisms underlying drug treatments. By employing metabolomics, researchers can comprehensively characterize metabolites within cells, organs, tissues, or biological fluids in a high-throughput manner, thereby gaining insights into physiological and pathological processes [10]. This technology not only facilitates the discovery of disease biomarkers but also enhances the understanding of drug mechanisms of action. Recently, numerous researchers have successfully integrated these methodologies to elucidate the mechanisms by which Qingfei Tongluo Plaster ameliorates respiratory syncytial virus pneumonia [7] and Qingyi Decoction treats acute pancreatitis [11].

Consequently, this study aims to initially identify and quantify the active constituents of DLD, integrating network pharmacology, experimental validation, and metabolomics research to preliminarily investigate the mechanism of DLD intervention in hyperthyroid kidney disease (HKD).

## 2. Materials and Methods

### 2.1. Drugs and Reagents

DLD was synthesized in the laboratory through a specific preparation method involving the combination of equal weights of Angelicae Sinensis Radix (20211201), Rehmanniae Radix (20210410), Rehmanniae Radix Praeparata (20201101), Scutellariae Radix (20210701), Coptidis Rhizoma (20200301), and Phellodendri Chinensis Cortex (20210701), along with a double quantity of Astragali Radix (20210101, all sourced from Jilin Guoan Pharmaceutical Co., Ltd.). Initially, the mixture underwent decoction with eight times its weight in distilled water for 1 h. This was followed by a second decoction with six times its weight in distilled water for an additional hour. The resultant solution was filtered through double-layer gauze, and the filtrates from both decoctions were combined. The extract was subsequently heated, concentrated, and subjected to vacuum freeze-drying at −50°C for 72 h to yield DLD freeze-dried powder, which was utilized for subsequent content determination and administration. The reference standards were purchased from Shanghai Yuanye Biotechnology Co., Ltd. and the China National Institute for Food and Drug Control, as detailed in the Supporting Information: Table S1. Both phosphoric acid (Tianjin Kemiou Chemical Reagent Co., Ltd.) and methanol (Fisher Scientific) were of HPLC grade.

### 2.2. Preparation of Sample Solutions and Standard Solutions

#### 2.2.1. Preparation of Standard Solutions

Reference standards “phellodendrine chloride, magnoflorine, ferulate, coptisine, epiberberine, jatrorrhizine, berberine, palmatine, baicalin, quercetin, wogonoside, and wogonin” were dissolved in methanol solutions to prepare the stock standard solutions with a concentration of 396.00, 92.50, 12.00, 97.50, 11.84, 100.00, 53.20, 272.43, 145.00, 60.00, 205.00, and 4.00 μg/ml.

#### 2.2.2. Preparation of Sample Solutions for the DLD

Accurately weigh 0.5 g of the sample and add precisely 25 mL of methanol. Reweigh the mixture, then sonicate for 30 min. Allow the solution to cool and adjust the weight as necessary. Shake the mixture thoroughly, filter it, and collect the filtrate. Accurately measure 1 mL of the filtrate and transfer it to a 5 mL volumetric flask, adjusting the volume with methanol to prepare the sample solution. This preparation method is consistently applied to all samples to ensure uniformity in subsequent analyses.

### 2.3. Chromatographic Condition

The high-performance liquid chromatography system utilized in this study was the Shimadzu LC-2030 Prominence-I series, which was equipped with an ultraviolet–visible (UV–vis) spectroscopy detector, a binary pump solvent management system with an integrated online degasser, a column oven, an automatic sampler, and a Lab Solutions workstation. Chromatographic separation was achieved using a ZORBAX Eclipse Plus C18 column (4.6 × 250 mm, 5 μm; Agilent), with the column temperature set at 30°C. The mobile phase consisted of methanol (A) and 0.2% phosphoric acid in water (B), utilizing a gradient elution method. The gradient elution program was structured as follows: from 0 to 20 min, increased from 18% to 26% A; at 35 min, increased from 26% to 40% A; at 50 min, increased from 40% to 56% A; at 60 min, increased from 56% to 60% A; at 70 min, increased from 60% to 100% A. The injection volume was 10 μL, with a flow rate of 1.0 mL/min, and the detection wavelength was set at 280 nm.

### 2.4. Network Pharmacology Analysis of HKD Intervened by DLD

We conducted a comprehensive search for the seven drug components of DLD using the Traditional Chinese Medicine Systematic Pharmacology Database and Analysis Platform (TCMSP, https://tcmsp-e.com/index.php). To identify active components, we applied screening criteria of oral availability ≥ 30% and drug-likeness ≥ 0.18. The corresponding target proteins of these active components were subsequently identified. These target protein names were then converted into gene names specific to verified human species using the UniProt database (https://www.uniprot.org/), thereby establishing the relevant targets of DLD.

In the OMMI (https://www.omim.org/), Drugbank (https://go.drugbank.com/), GeneCards (https://www.genecards.org/), and TTD (https://db.idrblab.net/ttd/) databases, “hyperthyroidism” and “kidney injury” were used as keywords to search, and the obtained genes were integrated and intersected, which were the target genes of HKD.

A Venn diagram analysis was utilized to ascertain the intersection targets of the active components of DLD and their correlation with HKD. These intersection targets were subsequently imported into the STRING database (https://cn.string-db.org/) to facilitate the construction and analysis of the protein–protein interaction (PPI) network. The analysis was conducted with the species parameter set to Homo sapiens and a minimum interaction score threshold established at medium confidence (0.400). The analysis results, formatted in TSV, were then imported into Cytoscape 3.10.2 software for visualization and further comprehensive analysis. Through topological analysis, key targets were identified and selected based on their potential significance within the context of HKD.

The intersection targets were submitted to the DAVID database (https://david.ncifcrf.gov/) for Gene Ontology (GO) functional analysis and Kyoto Encyclopedia of Genes and Genomes (KEGG) pathway enrichment analysis. The biological processes (BPs), cellular components (CCs), molecular functions (MFs), and signaling pathways associated with key targets were identified, revealing the potential underlying mechanisms of DLD in treating HKD. The results were then imported into the Wei Sheng Xin platform (https://www.bioinformatics.com.cn/) for visualization.

Utilizing Cytoscape 3.10.2 software, a “component-target-pathway” network diagram was constructed, primarily displaying the interactions between the main components of DLD, key targets, and the top 20 KEGG signaling pathways.

### 2.5. Study on the Mechanism of DLD in Intervening HKD

#### 2.5.1. Animals

Thirty-six Wistar rats, SPF grade, male, aged 6 to 8 weeks, were purchased from Liaoning Changsheng Biotechnology Co., Ltd., with the license number SCXK (Liao) 2020-0001. The animal experiments were approved by the Experimental Animal Ethics Committee of Changchun University of Chinese Medicine, with the approval number 2023170. Every effort was made to minimize the suffering of the animals.

#### 2.5.2. HKD Model and Drug Administration

The HKD model was induced as previously reported [12]. Briefly, the rats were modeled by daily intragastric administration of a suspension containing 75 mg/kg of thyroxine and 0.5 mg/kg of reserpine, once a day for 10 consecutive days. Thirty-six successfully modeled rats were randomly divided into six groups (*n* = 6), namely the blank control group (BC), the model group (Mod), the positive control group (treated with methimazole tablets, PC), and the low (L), medium (M), and high (H) dose groups of DLD (3.12, 6.24, and 12.48 g·kg^−1^·d^−1^, respectively). Rats in each treatment group were intragastrically administered the corresponding drug solution, while the model group and the normal group were given an equal volume of physiological saline intragastrically for 10 consecutive days.

#### 2.5.3. Observation of General State

Observe the following aspects of the rats: whether their fur is dry and yellowish, whether they are prone to hair loss, whether their ears, tail, and claws are red, whether the capillaries are engorged, and whether the stool is dry and hard. The first fecal pellet expelled by pulling the tail shall be taken as the standard for evaluating the state of the stool.

Weigh the rats and take their body measurements before modeling, before drug administration, and after the last drug administration.

Collect rat feces on modeling Days 0 and 10, and on the 10th day of drug administration, dry them in an environment of 105°C for 5 h, and determine the water content of the feces.

#### 2.5.4. Serum Hormone Level Determination

Rats were anesthetized by intraperitoneal injection of a 20% urethane solution, followed by blood collection from the abdominal aorta. Then, the blood is left to stand at room temperature for 1 h, followed by centrifugation at 5500 rpm for 10 min. The serum is collected, placed into cryovial tubes, and stored at −80°C. The levels of triiodothyronine (T3), tetraiodothyronine (T4), cyclic adenosine monophosphate (cAMP), cyclic guanosine monophosphate (cGMP), and thyroid-stimulating hormone (TSH) in the serum are measured using the enzyme-linked immunosorbent assay (ELISA) method (Jiangsu Meimian Industrial Co., Ltd).

#### 2.5.5. Histopathological Evaluation

At the time of euthanizing the rats, quickly collect the thyroid and kidney tissues and fix them in 4% paraformaldehyde. Then, embed the tissues in paraffin, further cut them into 5 μm thick sections, deparaffinize, and stain with hematoxylin–eosin (H&E), wash, mount, and observe under a light microscope.

#### 2.5.6. Determination of CAT, MDA, and SOD Levels in Kidney Tissue

We take another portion of the kidney tissue and place it into a cryovial tube, and then store it at −80°C. Combine chopped kidney tissue (10 mg) with 900 μL of phosphate-buffered saline (PBS) (containing protease inhibitors), homogenize on ice, and centrifuge the homogenate to obtain the supernatant, which is then set aside for use. Use different biochemical assay kits to detect the levels of catalase (CAT), malondialdehyde (MDA), and superoxide dismutase (SOD) (Jiangsu Meimian Industrial Co., Ltd).

#### 2.5.7. Western Blotting (WB)

The kidney tissues were homogenized using radio immunoprecipitation assay (RIPA) lysis buffer to extract total protein, and the protein concentration was determined using the bicinchoninic acid assay (BCA) method. Proteins were separated by 10% sodium dodecyl sulfate polyAcrylamide gel electrophoresis (SDS-PAGE) gel electrophoresis; the separated proteins were then transferred onto a polyvinylidene difluoride (PVDF) membrane and blocked with 5% nonfat milk at room temperature for 2 h. The membrane was then incubated overnight at 4°C with primary antibodies against nuclear factor erythroid 2-related factor 2 (Nrf2) (diluted 1:2000), heme oxygenase-1 (HO-1) (diluted 1:2000), beta-actin (diluted 1:2000) (Proteintech Group, Wuhan), phospho-phosphatidylinositol 3-kinase (p-PI3K) (diluted 1:1000), phosphor-protein kinase B (p-AKT) (diluted 1:1000) (Bioss, Beijing), PI3K (diluted 1:1000), and AKT (diluted 1:1000) (Selleck, American). The following day, the membrane was washed three times with Tris-buffered saline with Tween (TBST) for 10 min each, then incubated with horseradish peroxidase (HRP)-conjugated goat anti-rabbit secondary antibodies (diluted 1:5000) (Proteintech Group, Wuhan) at room temperature for 2 h. After washing the membrane three times with TBST for 10 min each, ECL chemiluminescent reagent was added, and the membrane was imaged using a fully automated chemiluminescence imaging analyzer.

### 2.6. Serum Metabolomics Research

#### 2.6.1. Sample Preparation

Serum samples initially stored at −80°C were subjected to a controlled thawing process involving a gradient temperature protocol, transitioning from −20°C to 4°C, followed by vortex mixing. For the purpose of protein precipitation, 100 μL of serum was mixed with 300 μL of prechilled methanol (−20°C) and incubated at −20°C for 30 min. The mixture was then centrifuged at 12,000 g (4°C) for 10 min, and the resulting supernatant was collected for LC–MS analysis.

Quality control (QC) samples were prepared by pooling equal volume aliquots from all experimental samples to ensure data reliability in metabolomic profiling. These QC samples were analyzed intermittently throughout the analytical sequence to monitor system stability and data reproducibility.

#### 2.6.2. UHPLC–MS/MS Analysis

Chromatographic separation was achieved using an ACQUITY UPLC H-Class PLUS system (Waters Corporation, Milford, MA, USA) interfaced with a Xevo G2-XS QTof mass spectrometer. The separation was performed on a ZORBAX RRHD Eclipse Plus C18 analytical column (2.1 × 100 mm, 1.8 μm; Agilent Technologies) maintained at 35°C. The mobile phase consisted of (A) 0.1% (v/v) aqueous formic acid and (B) acetonitrile, with a constant flow rate of 0.25 mL/min throughout the 16 min linear gradient elution program. The gradient elution program was as follows: 0–1 min, 5% B; 1–2 min, 5%–30% B; 2–3 min, 30%–40% B; 3–5 min, 40%–60% B; 5–10 min, 60% B; 10–11 min, 60%–90% B; 11–12 min, 90%; 12–13 min, 90%–45% B; 13–15 min, 45%–5% B; 15–16 min, 5% B. The sample injection volume was 5 μL.

The ion source operated in positive and negative electrospray ionization (ESI) modes. MSE scanning mode: Low energy (CE 4 eV) and high energy (CE 20–40 eV) MSE modes were used alternately during operation, argon (99.999%) was used as collision-induced dissociation gas, and nitrogen (> 99.5%) was used as desolvation and cone gas. The scanning range was m/z 50–1200. Scanning speed: 0.5 s for one scan.

#### 2.6.3. Data Processing and Metabolite Identification

Data preprocessing was conducted using Progenesis QI (v2.0, Nonlinear Dynamics, Newcastle, UK), encompassing peak alignment (retention time [RT] and mass-to-charge ratio [m/z] adjustment), peak detection, spectral deconvolution, and total intensity normalization. The preprocessed dataset was subsequently subjected to molecular formula prediction through systematic analysis of adduct ion patterns, molecular ion clusters, and characteristic fragment ions. Compound identification was performed through tandem interrogation of experimental data against domain-specific databases, complemented by in silico fragmentation pattern simulation. All identified species met stringent mass accuracy criteria, with parent ion mass errors and fragment ion deviations both constrained within 10 ppm thresholds.

#### 2.6.4. Identification and Enrichment Analysis of Differential Metabolites

Multivariate statistical analysis was systematically conducted, comprising unsupervised principal component analysis (PCA) for initial pattern exploration and supervised orthogonal partial least squares–discriminant analysis (OPLS–DA) for class discrimination modeling. Candidate differential metabolites were identified through dual-criteria filtering: variable importance in projection (VIP) scores exceeding 1.0 coupled with statistically significant thresholds (*p* < 0.05, Student's *t*-test). Structural annotation of these metabolites was achieved through cross-referencing with the Human Metabolome Database (HMDB, https://hmdb.ca/), while pathway enrichment analysis was executed via MetaboAnalyst 6.0 (https://www.metaboanalyst.ca/) using hypergeometric testing with false discovery rate (FDR) correction.

### 2.7. Integrated Analysis of Metabolomics and Network Pharmacology

Metabolites from metabolomics and targets of network pharmacology were jointly analyzed by the MetaboAnalyst database to construct a “metabolite-gene” network. In addition, the metabolite targets were obtained through the SEA database (https://sea.bkslab.org/), and the intersection targets of metabolites, DLD, and HKD were obtained through the Venn diagram, and the KEGG pathway enrichment analysis of the intersection targets was performed through the David database.

### 2.8. Data Processing and Analysis

GraphPad Prism 9.5 was used for statistical analysis of animal experimental data, and ImageJ software was employed for analyzing protein blotting data. Data were statistically analyzed using SPSS 25.0 software, with results expressed as the mean ± standard deviation ($\bar{x}$±s). One-way ANOVA was used for multiple group comparisons, and pairwise comparisons between groups were conducted using the LSD *t*-test. The significance level was set at *α* = 0.05.

## 3. Result

### 3.1. Determination of DLD Material Basis Content

Using HPLC, a total of 14 compounds were identified in DLD, including phellodendrine chloride, magnoflorine, ferulate, baicalin, wogonoside, baicalein, wogonin, epiberberine, coptisine, jatrorrhizine, palmatine, quercetin, ligustilide, and berberine. Furthermore, a method for the quantification of 12 of these components was developed and validated, as detailed in the supporting information (Tables S2–S6). Subsequently, the concentrations of these components were measured across 10 batches of DLD (Table 1).

### 3.2. Network Pharmacology Analysis of the Intervention of DLD in HKD

**Figure 1:**
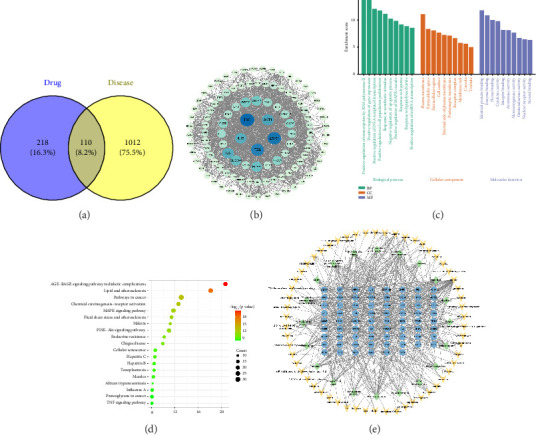
Network pharmacology analysis of DLD intervention HKD. Venn diagram (a), PPI network diagram (b), the node size is proportional to the degree value, GO enrichment analysis (c), KEGG pathway enrichment analysis (d), and network diagram of “components-targets-pathways” (e). Yellow arrows represent components, blue circles represent targets, and green diamonds represent signaling pathways.

A comprehensive analysis identified 91 primary active compounds of DLD through the TCMSP database. The associated target proteins were subsequently verified and refined using the UniProt database. Proteins without corresponding target genes were excluded, leading to the identification of 328 corresponding target genes. Additionally, 1517 target genes related to HKD were identified and combined with the target genes of the active compounds of DLD in Wei Sheng Xin to generate a Venn diagram. This analysis revealed 110 target genes that intersected between the active ingredients and the disease, indicating potential targets for HKD treatment (Figure 1(a)). The 110 intersected targets were then submitted to the STRING database to construct a PPI network, which was visualized and analyzed using Cytoscape Version 3.10.2, resulting in a network comprising 110 nodes and 1506 edges (Figure 1(b)).

GO and KEGG enrichment analysis was performed on 110 overlapping targets in the DAVID database. The screening condition was set to *p* < 0.05, and a total of 604 GO entries were enriched, of which 449 were BP, 64 were CC, and 91 were MF. In addition, 160 KEGG signaling pathways were enriched. In Figures 1(c) and 1(d), the top 10 GO entries and the top 20 KEGG pathways are listed in descending order by P-value. The signaling pathways involved in the potential targets of action are closely related to oxidative stress, cell proliferation, immune regulation, inflammatory response, and angiogenesis.

In order to further screen and validate the mechanism of action of the active ingredients of DLD in improving HKD, a “component-target-pathway” network was constructed by Cytoscape 3.10.2 (Figure 1(e)). The network revealed the ability of 59 compounds to exert biological activities by regulating 70 target proteins and 20 signaling pathways. These findings further support the notion that DLD treats HKD through multiple targets and pathways. Therefore, this network provides a theoretical basis for the screening of Q-markers and potential targets in DLD. The key targets showed close correlation with their associated signaling pathways.

### 3.3. Verification by Animal Experiments

#### 3.3.1. DLD Effectively Relieved HKD

The concurrent administration of thyroxine and reserpine effectively elicited characteristic symptoms and signs of hyperthyroidism in rats. In comparison to the blank control group, the rats in the model group exhibited noticeable emaciation, erect fur, hunched postures, increased difficulty in handling, frequent aggressive behavior, and significant weight loss. Additionally, their fecal matter was notably darker in color with a significantly reduced (all *p* < 0.01). Following treatment, all groups of rats demonstrated improved mental condition, lustrous fur, absence of erect fur and hunched postures, and no observed aggressive behavior, as compared to the model group. In terms of body weight, compared with the model group, there was a significant increase in body weight in the low, medium, and high dose groups of DLD (*p* < 0.05), and the level of increase approached that of the positive control group. Regarding feces, compared with the model group, there was no significant difference in the low-dose group of DLD; the high-dose group showed a highly significant increase in fecal water content (*p* < 0.01). This indicates that DLD can improve the condition of dry and hard stools in rats, with the high-dose group showing a more significant effect (Table 2).

**Figure 2:**
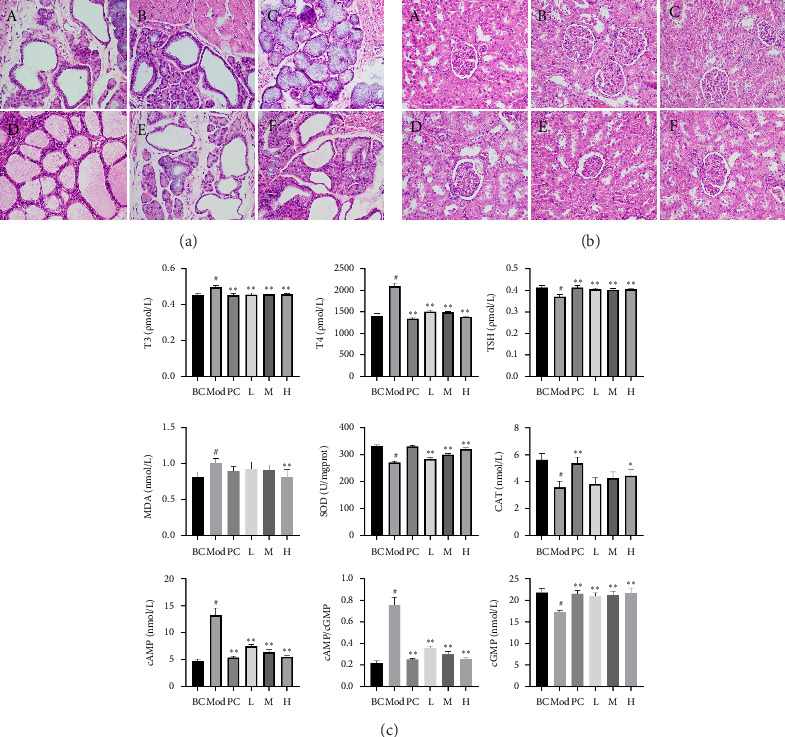
DLD effectively relieved HKD. Data are presented as mean ± SD; *n* = 6 per group. HE staining of thyroid tissue (magnification: 400×) (a), HE staining of renal tissue (magnification: 400×) (b), (A) is the BC, (B) is the Mod, (C) is the PC, (D) is the L, (E) is the M, and (F) is the H. Elisa results (c), compared with the BC, ^#^*p* < 0.05; compared with the Mod, ^∗^*p* < 0.05, ^∗∗^*p* < 0.01.

To evaluate the histopathological changes in the HKD, both the thyroid and kidneys were stained with HE. The results indicated that in the BC group, the thyroid tissue had clear and intact cellular structures (Figure 2(a)). In the Mod, there was disorder in cell arrangement, inflammatory infiltration between tissues, fusion phenomena, and the formation of irregularly large follicles. Relative to the Mod group, the treatment group showed reduced inflammatory infiltration, decreased follicular cell fusion, more uniform follicle size, fewer absorption vacuoles, and milder colloid defects. The kidney tissue in the BC had clear and intact cellular structures (Figure 2(b)). However, in the Mod group, the glomeruli exhibited congestion, enlarged capsular spaces, and inflammatory infiltration. Compared to the Mod group, the treatment group showed slightly reduced glomerular congestion, slightly enlarged capsular spaces, metabolic dilation of the renal tubules, and a small amount of fatty tissue infiltration. In the H group, there was mild glomerular congestion without significant enlargement of the capsular spaces and reduced dilation of the renal tubules.

Hormone level measurements (Figure 2(c)) indicated that the serum levels of T3 and T4 in the Mod group were significantly higher than in other groups (*p* < 0.01), while TSH was extremely significantly lower than in the BC group (*p* < 0.01). The content of cAMP and the ratio of cAMP/cGMP were significantly increased, and the content of cGMP was significantly decreased, indicating that the hyperthyroidism model was successfully established. The DLD showed a dose-dependent reversal of this process, with the H group effects approaching those of the positive control group, suggesting that DLD has a regulatory effect on thyroid-related hormones. The activity of SOD and CAT in renal tissue was significantly reduced, and MDA was markedly increased. Similarly, DLD showed a dose-dependent reversal of this process, with the H group effects approaching those of the PC. Overall, this indicates that DLD effectively inhibits HKD.

#### 3.3.2. The Treatment With DLD Suppressed the Expression of p-PI3K/PI3K, p-AKT/AKT, Nrf2, and HO-1

**Figure 3:**
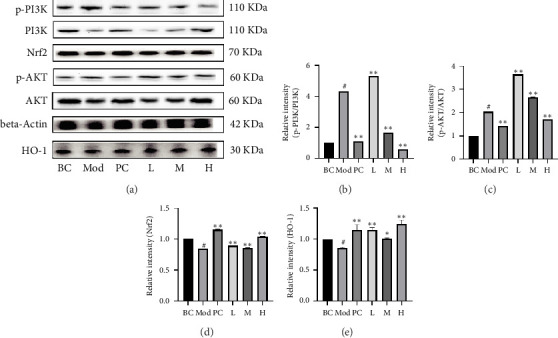
DLD inhibited PI3K and AKT phosphorylation, as well as increased Nrf2 and HO-1 expression. The protein expression of p-PI3K, PI3K, p-AKT, AKT, Nrf2, and HO-1 was determined using western blot (a). Relative intensity of p-PI3K/PI3K (b), p-AKT/AKT (c), Nrf2 (d), and HO-1 (e). Data are represent mean ± SD (*n* = 3). Compared with the BC, ^#^*p* < 0.05; compared with the Mod, ^∗^*p* < 0.05, ^∗∗^*p* < 0.01.

To ascertain the involvement of DLD participants in the regulation of key targets within the mitogen-activated protein kinase (MAPK) and PI3K-AKT signaling pathways, namely, Nrf2, HO-1, p-PI3K, PI3K, p-AKT, and AKT, WB was used to determine their expression in renal tissue. The study results indicated that Nrf2 and HO-1 were significantly down-regulated and p-PI3K/PI3K and p-AKT/AKT were significantly up-regulated in the Mod group compared with the BC group, suggesting that hyperthyroidism leads to significant oxidative damage in rat kidney tissues (Figure 3). Following pharmacological intervention, oxidative damage was alleviated, and the trend of protein expression was reversed.

### 3.4. DLD-Regulated Metabolic Levels

**Figure 4:**
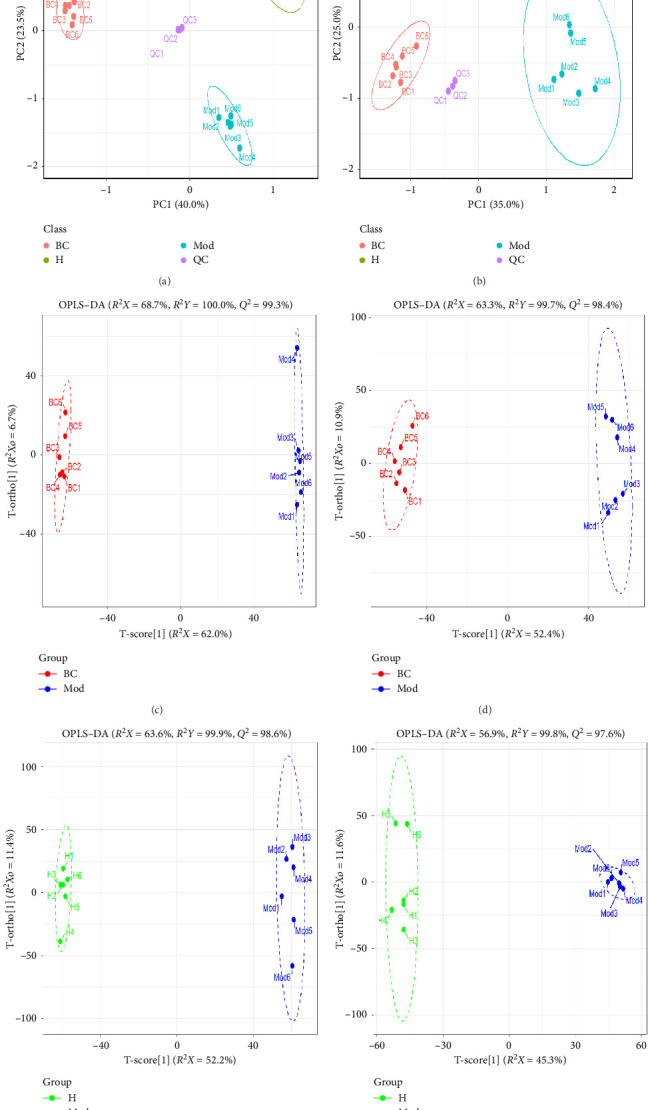
Multivariate statistical analysis of metabolomics. The PCA score in ESI + mode (a) and ESI − mode (b). The OPLS–DA score in ESI + mode (c) and ESI − mode (d) for the BC and Mod groups. The OPLS–DA score in ESI + mode (e) and ESI − mode (f) for the H and Mod groups.

To verify analytical reliability, QC samples were systematically monitored throughout the acquisition process. PCA revealed a tight clustering of QC samples (Figures 4(a) and 4(b)), confirming the stability of the instrumentation. Significant metabolic separation between the baseline control (BC) and model (Mod) groups was observed in both ionization modes, supporting model validity. Therapeutically, the H group demonstrated marked separation from the Mod group and showed convergence toward the BC group distribution, indicating an amelioration of symptoms.

OPLS–DA further discriminated metabolic profiles among groups. Clear separations between BC, Mod, and H groups were achieved in both ESI+ and ESI− modes (Figures 4(c), 4(d), 4(e), and 4(f)), with permutation tests confirming model validity (Q2 intercepts < 0). These results collectively establish DLD's therapeutic effect in rectifying metabolic disorders in HKD rats.

### 3.5. Metabolic Pathway Analysis

**Figure 5:**
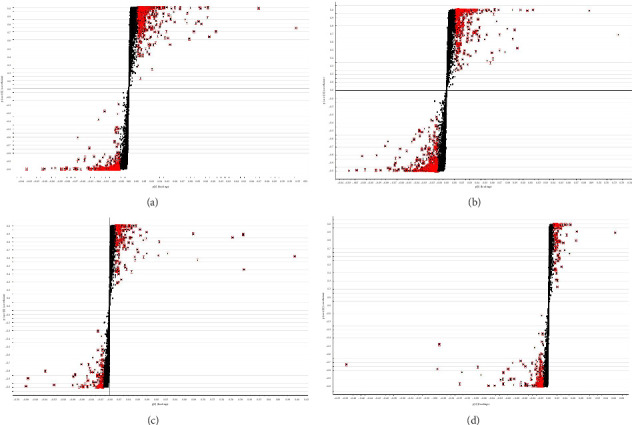
S-plot of different metabolites BC, Mod, and H group. S-plot of metabolites in BC with Mod in ESI− (a), S-plot of metabolites in Mod with H in ESI− (b), S-plot of metabolites in BC with Mod in ESI+ (c), S-plot of metabolites in Mod with H in ESI+ (d).

**Figure 6:**
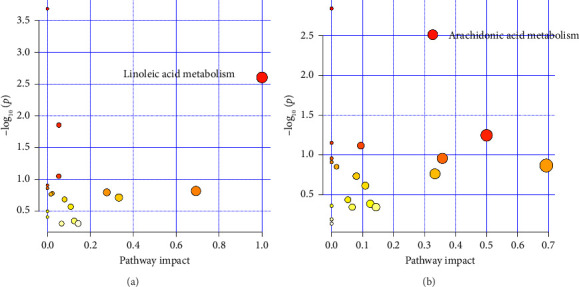
Enrichment analysis of differential metabolites. BC with Mod (a) and Mod with H (b).

In the OPLS–DA model, compounds with VIP > 1.0 and *p* value < 0.05 were screened as differential metabolites by *t*-test (Figure 5). There were 240 differential metabolites in the BC group and the Mod group and 244 differential metabolites in the Mod group and the H group. Metabolic network mapping was conducted using MetaboAnalyst 6.0 to characterize pathway-level perturbations in HKD progression and DLD therapeutic response in rat models. Pathways meeting the significance threshold (*p* < 0.05 and impact > 0.1) revealed a core dysregulated mechanism: linoleic acid metabolism. Post-DLD intervention, significant metabolic alterations were observed, particularly in the arachidonic acid metabolism pathway (Figure 6).

### 3.6. Potential Biomarker Screening

**Figure 7:**
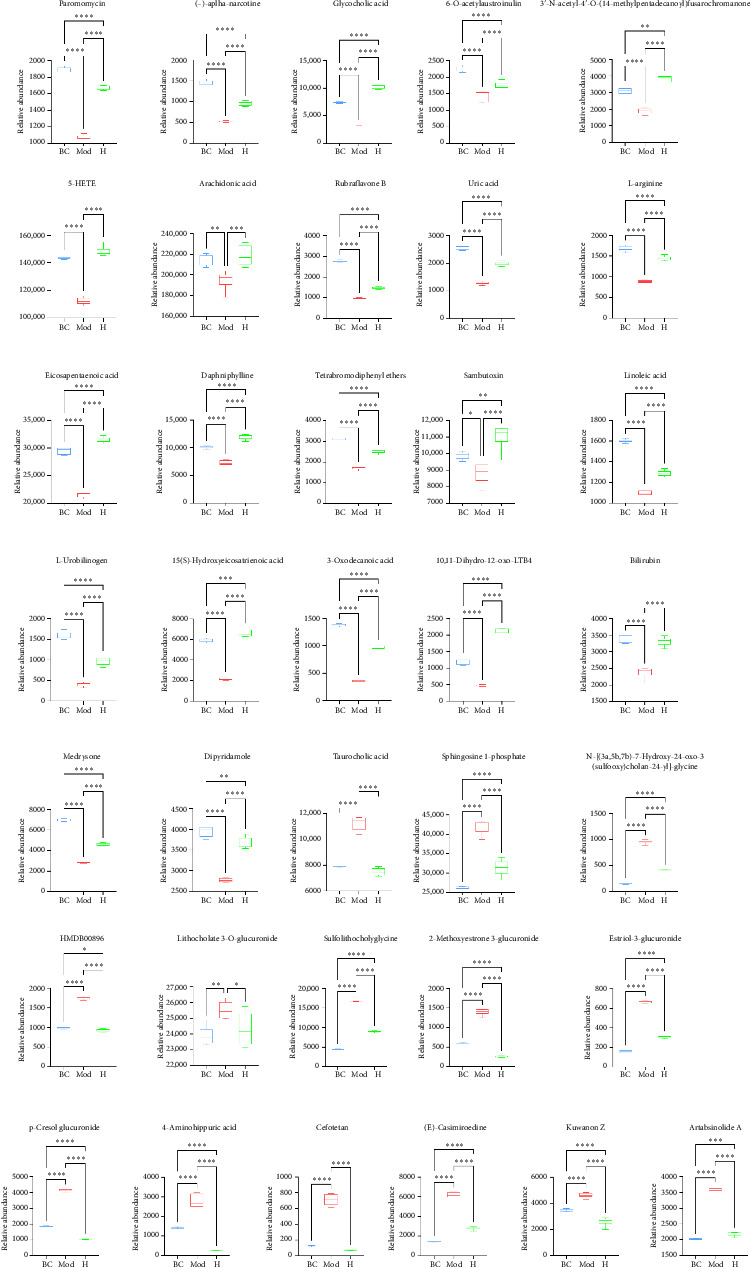
Potential biomarker changes in HKD with DLD treatment. Results are expressed as mean ± SD (*n* = 6). ^∗^*p* < 0.05, ^∗∗^*p* < 0.01, ^∗∗∗^*p* < 0.001, ^∗∗∗∗^*p* < 0.0001.

Metabolites that displayed significant upregulation or downregulation in the two comparison groups relative to the Mod group were identified as potential biomarkers (Figure 7). Specifically, 14 metabolites demonstrated significant upregulation, whereas 22 metabolites showed downregulation in the disease state. By integrating these findings with the pathway analysis presented in 3.5, which identified key metabolic pathways, linoleic acid, arachidonic acid and 5-HETE were ultimately selected as candidate biomarkers due to their critical roles in inflammatory and oxidative stress pathways.

### 3.7. Results of Integrated Analysis of Metabolomics and Network Pharmacology

**Figure 8:**
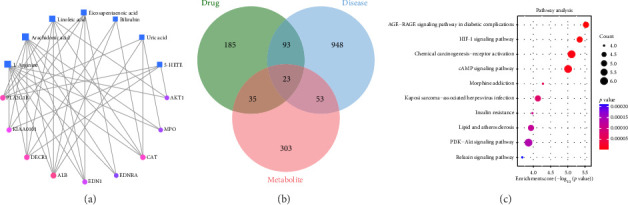
Integrated analysis of metabolomics and network pharmacology. “Metabolite-gene” network (a), Venn diagram (b), and KEGG pathway enrichment analysis (c).

To comprehensively elucidate the mechanism of DLD intervention in HKD, an interaction network was developed utilizing the aforementioned metabolomics and network pharmacology results (Figure 8(a)). This network identified nine key targets, namely PLA2G1B, KIAA0101, DECR1, ALB, EDN, EDNRA, CAT, MPO, and AKT1, as well as seven critical metabolites, including L-arginine, arachidonic acid, eicosapentaenoic acid, bilirubin, uric acid, and 5-HETE. In addition, a total of 414 targets (Figure 8(b)) were obtained from 36 metabolites, and 23 common targets were obtained after intersection with key targets of DLD and HKD. The results of KEGG enrichment again showed that the PI3K-AKT signaling pathway constitutes a pivotal pathway (Figure 8(c)).

## 4. Discussion

In this study, we effectively developed a multi-component content determination method for DLD, elucidated the potential mechanism of DLD intervention in HKD using network pharmacology, and validated these findings through pharmacological experiments. Subsequently, we conducted a metabolomics analysis to investigate the pathways associated with the occurrence of HKD and the intervention of DLD.

The network pharmacology analysis of DLD identified 91 active compounds that predominantly exert their effects by modulating targets or pathways associated with oxidative stress, cell proliferation, immune regulation, inflammatory responses, and angiogenesis, among others. To elucidate the core mechanisms, we constructed a “component-target-pathway” network diagram based on the top 20 pathways with the lowest *p* values. The principal pathways involved include the AGE-RAGE signaling pathway in diabetic complications, lipid and atherosclerosis, cancer pathways, chemical carcinogenesis—receptor activation, the MAPK signaling pathway, fluid shear stress and atherosclerosis, malaria, and the PI3K-AKT signaling pathway, among others. Notably, the AGE-RAGE signaling pathway in diabetic complications is intricately linked to hyperthyroidism-induced kidney damage and plays a pivotal role in the development of diabetic nephropathy. As our research group is actively investigating this pathway, we will abstain from discussing its implications in the current study. The cancer and malaria pathways are not pertinent to the diseases under investigation and thus will not be taken into consideration. The lipid and atherosclerosis and fluid shear stress and atherosclerosis pathways are closely related to lipid metabolism. Meanwhile, the PI3K-AKT signaling pathway is involved in diverse pathological processes such as lipid metabolism, accumulation of vulnerable plaques, and thrombosis formation [13]. Furthermore, chemical carcinogenesis—receptor activation is regulated by both the PI3K-AKT and MAPK signaling pathways. Therefore, this study focuses on analyzing the key proteins in these two pathways.

It is recognized and acknowledged that lipid peroxidation contributes to the excessive production and accumulation of reactive oxygen species (ROS), which subsequently induces oxidative stress, disrupts the endogenous antioxidant systems of mucosal cells, and results in cellular damage [14]. Under normal conditions, a balance is maintained between ROS and antioxidant enzymes (such as SOD, CAT, etc.). The cascade reactions triggered by oxidative stress can lead to the overproduction of ROS and the accumulation of MDA. MDA is a crucial marker of ROS peroxidation because it is a major end product of lipid peroxidation and can directly reflect the extent of peroxidation reactions in the body [15]. When oxidative stress takes place, the antioxidant defense system also actively functions to clear ROS, defend against intracellular oxidative stress, and safeguard the body [16], which is manifested in the fact that ROS activate the phosphorylation of PI3K and AKT through various mechanisms [17]. Moreover, the MAPK signaling pathway is involved in the mechanisms of oxidative stress and inflammatory responses. Nrf2 and HO-1 serve as critical downstream effectors within this pathway, and their cascade reactions play a pivotal role in the body's anti-inflammatory and antioxidant defenses [18, 19]. Nrf2 functions as a principal transcriptional regulator in cellular defense against oxidative damage and is capable of significantly enhancing endogenous antioxidant responses [20]. Consequently, it represents a crucial therapeutic target for mitigating oxidative stress-related damage in a variety of diseases. The activation of Nrf2 results in its translocation to the nucleus, which subsequently initiates the transcriptional activation of a series of downstream antioxidant protective genes, including HO-1, SOD, and CAT [21]. HO-1, recognized as a stress-inducible enzyme, serves as a reliable antioxidant and cellular protector; it catalyzes the breakdown of heme into equimolar amounts of biliverdin, ferrous iron, and carbon monoxide [22]. In this study, in the drug-induced rats, the MDA levels in kidney tissue exhibited a substantial increase, while the concentrations of SOD and CAT showed a marked decrease, indicating enhanced oxidative stress and lipid peroxidation. Additionally, it led to the activation of the expression and phosphorylation of PI3K and AKT, along with the inhibition of the expression of Nrf2 and HO-1. DLD demonstrated remarkable antioxidant activity, as high-dose intervention with DLD significantly reduced MDA levels and markedly increased SOD and CAT levels, thereby reversing the observed trends in protein expression.

To systematically elucidate the therapeutic effects of DLD, we utilized serum metabolomics to characterize endogenous metabolic disturbances within the HKD model and assess treatment outcomes. The findings suggested that the metabolism of linoleic acid and arachidonic acid may represent critical metabolic pathways implicated in both disease pathogenesis and drug efficacy. Furthermore, linoleic acid, arachidonic acid, and 5-HETE were identified as potential biomarkers. Subsequent integration of these findings with network pharmacology analysis further validated the reliability of the biomarker selection and confirmed the accuracy of the pathway screening within the context of network pharmacology. Based on these findings, the hyperthyroid rat model demonstrated a significant depletion of essential polyunsaturated fatty acids (PUFAs), including linoleic acid, arachidonic acid, and 5-hydroxyeicosatetraenoic acid (5-HETE), paralleled by significant oxidative stress imbalance. This metabolic-oxidative coupling manifested as decreased antioxidant capacity (the levels of SOD and CAT decreased) and exacerbated lipid peroxidation (the levels of SOD increased), consistent with thyrotoxicosis-induced metabolic reprogramming [23]. Mechanistically, the observed PUFA depletion correlated with enhanced PI3K/AKT pathway activation, suggesting intensified fatty acid β-oxidation through phosphorylation-mediated catabolic regulation [24]. Notably, arachidonate derivatives like 5-HETE serve dual roles as metabolic intermediates and redox signaling modulators. 5-Lipoxygenase (5-LOX)-generated 5-HETE undergoes enzymatic conversion to 5-oxo-ETE, a potent Nrf2 activator that promotes HO-1 expression via antioxidant response element (ARE) binding [25]. The observed deficiency in 5-HETE likely disrupts the protective axis, compromising the efficiency of Nrf2 nuclear translocation and subsequent induction of HO-1, which further leads to oxidative damage. Therapeutic intervention with DLD effectively reversed these pathological cascades. These multitarget effects align with recent findings that 5-LOX metabolites regulate endothelial Nrf2 activation through 5-HEPE-mediated epigenetic modifications [26]. This suggests that the therapeutic efficacy of DLD may involve the preservation of PUFA-derived signaling lipids, which are essential for maintaining cellular redox equilibrium under thyrotoxic stress.

Despite utilizing network pharmacological analysis, metabolomics, and in vivo experiments to explore the pharmacological mechanisms underlying DLD treatment in HKD, our study has been subject to several limitations. First, the compound components identified through network pharmacology were predicted using TCMSP, which lacks advanced in vivo transitional component analysis. Second, further in vitro empirical evidence is necessary to elucidate the complex mechanisms of DLD in the treatment of HKD. Finally, the QC methods and quality markers for DLD have not yet been comprehensively established.

## Figures and Tables

**Table 1:** The contents of these components in 10 batches of DLD.

Batch	Contents (mg)											
	Phellodendrine chloride	Magnoflorine	Ferulic	Coptisine	Epiberberine	Jatrorrhizine	Berberine	Palmatine	Baicalin	Quercetin	Wogonoside	Wogonin
1	6.13	1.13	0.30	1.83	0.18	0.87	4.97	1.39	7.62	2.23	2.33	0.10
2	5.54	0.74	0.43	1.95	0.19	1.03	5.10	1.55	9.68	2.60	2.98	0.04
3	5.59	0.76	0.43	2.22	0.22	0.97	5.39	1.52	10.00	2.45	2.75	0.05
4	5.58	0.68	0.28	2.30	0.19	0.94	5.95	1.39	8.92	2.15	2.36	0.05
5	4.40	0.60	0.33	1.96	0.17	0.91	4.83	1.35	7.69	1.97	2.27	0.05
6	6.16	0.79	0.34	2.04	0.19	0.94	5.88	1.45	8.32	2.09	2.19	0.05
7	5.12	0.72	0.48	1.92	0.17	1.01	5.25	1.45	9.56	2.16	2.65	0.06
8	4.88	0.71	0.23	1.99	0.17	1.01	5.62	1.37	10.46	2.25	2.74	0.07
9	4.44	0.59	0.18	1.94	0.16	1.01	4.81	1.39	7.94	1.67	2.10	0.06
10	4.20	0.69	026	1.80	0.15	1.00	4.95	1.46	8.39	1.78	2.02	0.05

**Table 2:** Changes in body weight and fecal water content (*n* = 6).

Group	Changes in body weight (g)			Fecal water content (%)		
	Day 0	Day 10	Day 20	Day 0	Day 10	Day 20
BC	0	91.26 ± 9.43	20.49 ± 1.41	66.49 ± 4.98	62.59 ± 2.35	64.51 ± 1.24
Mod	0	67.16 ± 8.75^##^	13.67 ± 1.62^##^	71.38 ± 3.19	38.82 ± 2.45^##^	51.81 ± 2.39^##^
PC	0	65.83 ± 5.44^##^	22.07 ± 2.98^∗∗^	67.56 ± 5.69	39.26 ± 3.21^##^	63.74 ± 3.49^∗∗^
L	0	66.79 ± 8.22^##^	15.44 ± 2.35^##∗^	64.11 ± 4.34	37.43 ± 2.15^##^	54.13 ± 2.33^##^
M	0	62.13 ± 9.48^##^	17.88 ± 1.86^∗∗^	63.78 ± 5.32	37.93 ± 3.10^##^	55.33 ± 2.22^##∗^
H	0	59.22 ± 10.68^##^	20.48 ± 1.88^∗∗^	65.75 ± 4.72	38.96 ± 2.49^##^	58.28 ± 1.93^#∗∗^

*Note:* Data are presented as mean ± SD; *n* = 6 per group. Comparison with the model group: ^∗^*p* < 0.05, ^∗∗^*p* < 0.01; comparison with the blank control group: ^#^*p* < 0.05, ^##^*p* < 0.01.

## Data Availability

The datasets used and analyzed during the current study are available from the corresponding author on reasonable request.

## Supporting Information

Table S1: Reference standards information.

Table S2: High-performance liquid chromatography methodology—linearity test.

Table S3: High-performance liquid chromatography methodology—precision test.

Table S4: High-performance liquid chromatography methodology—repeatability test.

Table S5: High-performance liquid chromatography methodology—stability test.

Table S6: High-performance liquid chromatography methodology—accuracy test.

Table S7: Information of 91 components in DLD.
