# Supplementary material for: Elucidating the Therapeutic Mechanism of Danggui Liuhuang Decoction in Hyperthyroid Kidney Disease: An Integration of Network Pharmacology and Metabolomics
Source: Int J Endocrinol. 2025 Sep 28;2025:5513418. doi: 10.1155/ije/5513418 (PMC12497527; doi:10.1155/ije/5513418)
Supplement: Supporting Information — Additional supporting information can be found online in the Supporting Information section. [file 5513418.f1.docx]

1. Linearity

The mixed standard solution was accurately taken (Table S1), diluted in different proportions and injected for determination. The standard curve was drawn with the injection volume as the abscissa and the peak area as the ordinate, and the regression equation was calculated. The results are shown in Table S2.

Table S1 Reference standards

| No. | Name | Batch number | Purity | Factories |
| --- | --- | --- | --- | --- |
| 1 | Phellodendrine chloride | W11A9Z58574 | 98% | Shanghai yuanye Bio-Technology Co., Ltd |
| 2 | Magnoflorine | T21S11C125202 | 98% |  |
| 3 | Ferulate | W27S9Z71290 | 98% |  |
| 4 | Baicalin | 110715-201720 | 93.5% |  |
| 5 | Wogonoside | 112002-201702 | 98.5% |  |
| 6 | Baicalein | 111595-201607 | 98.5% |  |
| 7 | Wogonin | 111873-201704 | 99.8% |  |
| 8 | Epiberberine | W11A9Z58574 | 98% |  |
| 9 | Coptisine | T21S11C125202 | 98% |  |
| 10 | Jatrorrhizine | W22A11467 | 98% |  |
| 11 | Palmatine | W11A12782 | 98% |  |
| 12 | Quercetin | 11453-201805 | 98% |  |
| 13 | Ligustilide | B20492 | 98% |  |
| 14 | Berberine | 110713-201212 | 86.7% | National Institutes for Food and Drug Control |

Table S2 Linearity test results

| Name | Linearity | R | Range (μg/mL) |
| --- | --- | --- | --- |
| Phellodendrine chloride | y = 122,228.2438 x - 66.4657 | 0.9999 | 0.65-396.00 |
| Magnoflorine | y = 1,552,609.0492 x - 7,634.1180 | 0.9996 | 0.15-92.50 |
| Ferulate | y = 3,503,157.7748 x - 417.4966 | 0.9999 | 0.05-30.00 |
| Coptisine | y = 2,269,090.1662 x - 724.2537 | 0.9999 | 0.16-97.50 |
| Epiberberine | y = 24,566,401.2415 x + 455.6487 | 0.9999 | 0.02-11.84 |
| Jatrorrhizine | y = 3,199,680.6328 x + 1,840.9812 | 0.9999 | 0.16-100.00 |
| Berberine | y = 5,497,469.8115 x + 2,978.7836 | 0.9999 | 0.09-53.20 |
| Palmatine | y = 3,740,641.5930 x + 5,352.2739 | 0.9999 | 0.45-272.43 |
| Baicalin | y = 4,499,437.3001 x - 68,107.2478 | 0.9999 | 0.594-362.50 |
| Quercetin | y = 1,442,859.9772 x - 836.4952 | 0.9999 | 0.246-150.00 |
| Wogonoside | y = 3,769,731.0638 x - 26,375.7147 | 0.9999 | 0.840-512.50 |
| Wogonin | y = 9,058,121.3951 x - 258.6888 | 0.9999 | 0.016-10.00 |

2. Precision

10 μl of the sample solution was accurately sucked, and the sample was injected repeatedly for 6 times. The RSD values of each peak area were calculated (%). The results are shown in Table S3, and the RSD values are all less than 3 %, indicating that the instrument has good precision.

Table S3 Precision test results

| Name | 1 | 2 | 3 | 4 | 5 | 6 | Average | RSD (%) |
| --- | --- | --- | --- | --- | --- | --- | --- | --- |
| Phellodendrine chloride | 15504 | 15167 | 15498 | 15153 | 15838 | 15586 | 15458 | 1.69 |
| Magnoflorine | 31544 | 31080 | 31559 | 31295 | 31340 | 31400 | 31370 | 0.57 |
| Ferulate | 10199 | 10217 | 10147 | 10383 | 10062 | 10662 | 10278 | 2.10 |
| Coptisine | 97615 | 96319 | 93422 | 94896 | 96153 | 98061 | 96078 | 1.79 |
| Epiberberine | 107281 | 102994 | 99365 | 100799 | 102700 | 104083 | 102870 | 2.66 |
| Jatrorrhizine | 101691 | 97433 | 94780 | 95711 | 96768 | 98008 | 97399 | 2.47 |
| Berberine | 817277 | 809383 | 799466 | 811320 | 808044 | 814663 | 810026 | 0.76 |
| Palmatine | 168857 | 162212 | 158945 | 161057 | 160921 | 162228 | 162370 | 2.09 |
| Baicalin | 1468289 | 1409969 | 1395656 | 1415027 | 1408837 | 1422840 | 1420103 | 1.78 |
| Quercetin | 106086 | 105097 | 104705 | 106099 | 105631 | 107457 | 105846 | 0.91 |
| Wogonoside | 323213 | 319918 | 317081 | 321370 | 319894 | 322257 | 320622 | 0.68 |
| Wogonin | 35026 | 35237 | 35631 | 34249 | 34594 | 34143 | 34813 | 1.68 |

3. Repeatability

Take the same batch of samples, prepare 6 groups of parallel samples according to the above “2.2.2” method, and inject the samples. The test results are shown in Table S4, the average contents of phellodendrine chloride, magnoflorine, ferulate, coptisine, epiberberine, jatrorrhizine, berberine, palmatine, baicalin, quercetin, wogonoside and wogonin were 3.4051, 0.5418, 0.0722, 1.0554, 0.1046, 0.7691, 3.6937, 1.0891, 7.8577, 1.8661, 2.0870 and 0.0989 mg / g, respectively. The RSD values were 2.09, 1.40, 1.76, 0.97, 0.80, 0.78, 0.46, 0.43, 2.88, 1.97, 1.83, 2.28 and 1.71, respectively. All of them were less than 3.0 %, indicating that the method had good repeatability.

Table S4 Repeatability test result

| Name | Contents/ (mg/g) | | | | | | Average | RSD (%) |
| --- | --- | --- | --- | --- | --- | --- | --- | --- |
|  | 1 | 2 | 3 | 4 | 5 | 6 |  |  |
| Phellodendrine chloride | 3.4051 | 3.2750 | 3.2378 | 3.2686 | 3.2229 | 3.2262 | 3.4051 | 2.09 |
| Magnoflorine | 0.5418 | 0.5369 | 0.5346 | 0.5522 | 0.5334 | 0.5319 | 0.5418 | 1.40 |
| Ferulate | 0.0722 | 0.0758 | 0.0743 | 0.0740 | 0.0730 | 0.0727 | 0.0722 | 1.76 |
| Coptisine | 1.0554 | 1.0666 | 1.0480 | 1.0746 | 1.0599 | 1.0725 | 1.0554 | 0.97 |
| Epiberberine | 0.1046 | 0.1045 | 0.1038 | 0.1058 | 0.1047 | 0.1059 | 0.1046 | 0.80 |
| Jatrorrhizine | 0.7691 | 0.7685 | 0.7726 | 0.7817 | 0.7811 | 0.7794 | 0.7691 | 0.78 |
| Berberine | 3.6937 | 3.6460 | 3.6845 | 3.6897 | 3.6797 | 3.6761 | 3.6937 | 0.46 |
| Palmatine | 1.0891 | 1.0826 | 1.0889 | 1.0952 | 1.0950 | 1.0914 | 1.0891 | 0.43 |
| Baicalin | 7.8577 | 8.3217 | 7.9215 | 8.3741 | 7.9595 | 8.3046 | 7.8577 | 2.88 |
| Quercetin | 1.8661 | 1.9009 | 1.8925 | 1.9032 | 1.8057 | 1.8875 | 1.8661 | 1.97 |
| Wogonoside | 2.0870 | 2.1553 | 2.0727 | 2.1585 | 2.0959 | 2.1495 | 2.0870 | 1.83 |
| Wogonin | 0.0989 | 0.0997 | 0.0951 | 0.0985 | 0.0970 | 0.0971 | 0.0989 | 2.28 |

4. Stability

The sample solution was taken and injected at 0, 2, 4, 6, 8, 10, 12 and 24 h according to the chromatographic conditions under “2.2.3”. The RSD value of the peak area was calculated. The results showed that the RSD value of the peak area contained in Danggui Liuhuang Decoction was less than 3.0 %, indicating that the sample was stable within 24 h. The results are shown in Table S5.

Table S5 Stability test result

| Name | 0 | 2 | 4 | 6 | 8 | 10 | 12 | 24 | Average | RSD (%) |
| --- | --- | --- | --- | --- | --- | --- | --- | --- | --- | --- |
| Phellodendrine chloride | 15183 | 15345 | 15399 | 15344 | 15815 | 15704 | 15753 | 16344 | 15727 | 2.28 |
| Magnoflorine | 30844 | 30474 | 31336 | 30113 | 30181 | 30230 | 31021 | 30886 | 30628 | 1.69 |
| Ferulate | 10554 | 10469 | 10204 | 10499 | 10000 | 10616 | 10208 | 10297 | 10304 | 2.16 |
| Coptisine | 95091 | 94325 | 94775 | 95566 | 95834 | 96785 | 97393 | 95839 | 96032 | 0.96 |
| Epiberberine | 97100 | 101773 | 101968 | 102566 | 102559 | 103160 | 104207 | 102729 | 102865 | 0.74 |
| Jatrorrhizine | 98433 | 98538 | 98602 | 98720 | 99095 | 99688 | 101858 | 97063 | 99171 | 1.59 |
| Berberine | 802312 | 805355 | 813433 | 803352 | 810992 | 813460 | 815006 | 816424 | 812111 | 0.57 |
| Palmatine | 161492 | 161070 | 162001 | 160915 | 161807 | 162910 | 166041 | 163607 | 162880 | 1.11 |
| Baicalin | 1400411 | 1393372 | 1408134 | 1388949 | 1403012 | 1407320 | 1460324 | 1423324 | 1415177 | 1.75 |
| Quercetin | 105022 | 102193 | 103242 | 101566 | 102446 | 102792 | 104657 | 106593 | 103549 | 1.74 |
| Wogonoside | 317078 | 316420 | 317420 | 313292 | 318872 | 319744 | 321305 | 322803 | 318906 | 1.04 |
| Wogonin | 35527 | 33320 | 32368 | 33509 | 33445 | 33334 | 32922 | 32830 | 33068 | 1.34 |

5. Accuracy

The appropriate amount of Danggui Liuhuang Decoction was added to each reference substance according to the proportion of 100 % of the component content. The test solution was prepared by the method under “2.2.2”, and the sample was injected for determination. The average recovery rate and RSD value of each component were calculated. The average recoveries of phellodendrine chloride, magnoflorine, ferulate, coptisine, epiberberine, jatrorrhizine, berberine, palmatine, baicalin, quercetin, wogonoside and wogonin were 100.35 %, 100.69 %, 100.97 %, 101.11 %, 100.54 %, 100.14 %, 99.67 %, 99.39 %, 99.66 %, 101.46 %, 100.06 %, 14.37 % and 99.24 %, respectively. The RSD values were 2.60 %, 0.97 %, 2.39 %, 2.82 %, 2.99 %, 2.64 %, 2.47 %, 2.49 %, 2.64 %, 2.20 %, 2.99 %, 1.93 %, respectively, which were all less than 3.0 %, indicating that the accuracy was good and the method was reliable. The results are shown in Table S6.

Table S6 Accuracy test result

| Name | No. | Sample weight (g) | Original quantity (mg) | Adding quantity (mg) | Measured (mg) | Recovery (%) | Average (%) | RSD (%) |
| --- | --- | --- | --- | --- | --- | --- | --- | --- |
| Phellodendrine chloride | 1 | 0.2498 | 0.8175 | 0.9568 | 1.7841 | 101.02 | 100.35 | 2.60 |
|  | 2 | 0.2502 | 0.8188 | 0.9568 | 1.7515 | 97.48 |  |  |
|  | 3 | 0.2503 | 0.8191 | 0.9568 | 1.8012 | 102.64 |  |  |
|  | 4 | 0.2505 | 0.8198 | 0.9568 | 1.7649 | 98.77 |  |  |
|  | 5 | 0.2499 | 0.8178 | 0.9568 | 1.8125 | 103.96 |  |  |
|  | 6 | 0.2495 | 0.8165 | 0.9568 | 1.7564 | 98.23 |  |  |
| Magnoflorine | 1 | 0.2498 | 0.1345 | 0.1534 | 0.2888 | 100.6 | 100.69 | 0.97 |
|  | 2 | 0.2502 | 0.1347 | 0.1534 | 0.287 | 99.25 |  |  |
|  | 3 | 0.2503 | 0.1348 | 0.1534 | 0.2907 | 101.66 |  |  |
|  | 4 | 0.2505 | 0.1349 | 0.1534 | 0.2911 | 101.85 |  |  |
|  | 5 | 0.2499 | 0.1346 | 0.1534 | 0.288 | 100.02 |  |  |
|  | 6 | 0.2495 | 0.1343 | 0.1534 | 0.2889 | 100.73 |  |  |
| Ferulate | 1 | 0.2498 | 0.0184 | 0.0224 | 0.0408 | 99.85 | 100.97 | 2.39 |
|  | 2 | 0.2502 | 0.0184 | 0.0224 | 0.0408 | 99.81 |  |  |
|  | 3 | 0.2503 | 0.0184 | 0.0224 | 0.0419 | 104.7 |  |  |
|  | 4 | 0.2505 | 0.0185 | 0.0224 | 0.0416 | 103.24 |  |  |
|  | 5 | 0.2499 | 0.0184 | 0.0224 | 0.0405 | 98.53 |  |  |
|  | 6 | 0.2495 | 0.0184 | 0.0224 | 0.0407 | 99.71 |  |  |
| Coptisine | 1 | 0.2498 | 0.2655 | 0.2887 | 0.5556 | 100.5 | 101.11 | 2.82 |
|  | 2 | 0.2502 | 0.2659 | 0.2887 | 0.5635 | 103.08 |  |  |
|  | 3 | 0.2503 | 0.266 | 0.2887 | 0.5664 | 104.05 |  |  |
|  | 4 | 0.2505 | 0.2662 | 0.2887 | 0.5636 | 103 |  |  |
|  | 5 | 0.2499 | 0.2656 | 0.2887 | 0.553 | 99.56 |  |  |
|  | 6 | 0.2495 | 0.2652 | 0.2887 | 0.5436 | 96.44 |  |  |
| Epiberberine | 1 | 0.2498 | 0.0262 | 0.0285 | 0.0559 | 104.3 | 100.54 | 2.99 |
|  | 2 | 0.2502 | 0.0262 | 0.0285 | 0.0551 | 101.37 |  |  |
|  | 3 | 0.2503 | 0.0263 | 0.0285 | 0.0553 | 101.98 |  |  |
|  | 4 | 0.2505 | 0.0263 | 0.0285 | 0.0551 | 101.25 |  |  |
|  | 5 | 0.2499 | 0.0262 | 0.0285 | 0.0544 | 98.8 |  |  |
|  | 6 | 0.2495 | 0.0262 | 0.0285 | 0.0534 | 95.56 |  |  |
| Jatrorrhizine | 1 | 0.2498 | 0.1937 | 0.1923 | 0.3949 | 104.61 | 100.14 | 2.64 |
|  | 2 | 0.2502 | 0.194 | 0.1923 | 0.3868 | 100.27 |  |  |
|  | 3 | 0.2503 | 0.1941 | 0.1923 | 0.3888 | 101.23 |  |  |
|  | 4 | 0.2505 | 0.1942 | 0.1923 | 0.3857 | 99.54 |  |  |
|  | 5 | 0.2499 | 0.1938 | 0.1923 | 0.3823 | 98.06 |  |  |
|  | 6 | 0.2495 | 0.1935 | 0.1923 | 0.3803 | 97.14 |  |  |
| Berberine | 1 | 0.2498 | 0.9188 | 1.005 | 1.9601 | 103.6 | 99.67 | 2.47 |
|  | 2 | 0.2502 | 0.9203 | 1.005 | 1.9085 | 98.33 |  |  |
|  | 3 | 0.2503 | 0.9207 | 1.005 | 1.9346 | 100.88 |  |  |
|  | 4 | 0.2505 | 0.9214 | 1.005 | 1.9158 | 98.94 |  |  |
|  | 5 | 0.2499 | 0.9192 | 1.005 | 1.923 | 99.88 |  |  |
|  | 6 | 0.2495 | 0.9177 | 1.005 | 1.8862 | 96.36 |  |  |
| Palmatine | 1 | 0.2498 | 0.2724 | 0.2798 | 0.5601 | 102.85 | 99.39 | 2.49 |
|  | 2 | 0.2502 | 0.2728 | 0.2798 | 0.55 | 99.08 |  |  |
|  | 3 | 0.2503 | 0.2729 | 0.2798 | 0.5544 | 100.59 |  |  |
|  | 4 | 0.2505 | 0.2731 | 0.2798 | 0.551 | 99.31 |  |  |
|  | 5 | 0.2499 | 0.2725 | 0.2798 | 0.5502 | 99.26 |  |  |
|  | 6 | 0.2495 | 0.272 | 0.2798 | 0.5386 | 95.26 |  |  |
| Baicalin | 1 | 0.2498 | 2.0292 | 2.45 | 4.4162 | 97.43 | 99.66 | 2.64 |
|  | 2 | 0.2502 | 2.0324 | 2.45 | 4.5141 | 101.29 |  |  |
|  | 3 | 0.2503 | 2.0332 | 2.45 | 4.3991 | 96.57 |  |  |
|  | 4 | 0.2505 | 2.0349 | 2.45 | 4.5574 | 102.96 |  |  |
|  | 5 | 0.2499 | 2.03 | 2.45 | 4.4318 | 98.03 |  |  |
|  | 6 | 0.2495 | 2.0267 | 2.45 | 4.5174 | 101.66 |  |  |
| Quercetin | 1 | 0.2498 | 0.4686 | 0.52 | 0.9813 | 98.59 | 101.46 | 2.20 |
|  | 2 | 0.2502 | 0.4694 | 0.52 | 1.0102 | 104.01 |  |  |
|  | 3 | 0.2503 | 0.4696 | 0.52 | 0.996 | 101.24 |  |  |
|  | 4 | 0.2505 | 0.4699 | 0.52 | 1.0119 | 104.23 |  |  |
|  | 5 | 0.2499 | 0.4688 | 0.52 | 0.9907 | 100.35 |  |  |
|  | 6 | 0.2495 | 0.4681 | 0.52 | 0.99 | 100.37 |  |  |
| Wogonoside | 1 | 0.2498 | 0.5295 | 0.73 | 1.2454 | 98.06 | 100.06 | 2.99 |
|  | 2 | 0.2502 | 0.5304 | 0.73 | 1.2852 | 103.4 |  |  |
|  | 3 | 0.2503 | 0.5306 | 0.73 | 1.2372 | 96.79 |  |  |
|  | 4 | 0.2505 | 0.531 | 0.73 | 1.2869 | 103.55 |  |  |
|  | 5 | 0.2499 | 0.5297 | 0.73 | 1.2423 | 97.61 |  |  |
|  | 6 | 0.2495 | 0.5289 | 0.73 | 1.2656 | 100.92 |  |  |
| Wogonin | 1 | 0.2498 | 0.0244 | 0.0247 | 0.049 | 99.54 | 99.24 | 1.93 |
|  | 2 | 0.2502 | 0.0244 | 0.0247 | 0.0493 | 100.7 |  |  |
|  | 3 | 0.2503 | 0.0245 | 0.0247 | 0.0484 | 96.78 |  |  |
|  | 4 | 0.2505 | 0.0245 | 0.0247 | 0.0496 | 101.54 |  |  |
|  | 5 | 0.2499 | 0.0244 | 0.0247 | 0.0491 | 99.79 |  |  |
|  | 6 | 0.2495 | 0.0244 | 0.0247 | 0.0484 | 97.11 |  |  |

Table S7 Information of 91 components in DLD

| No. | Mol ID | Name | OB% | DL | Rescoure |
| --- | --- | --- | --- | --- | --- |
| 1 | MOL000358 | beta-sitosterol | 36.91 | 0.75 | ANGELICAE SINENSIS RADIX  SCUTELLARIAE RADIX  PHELLODENDRI CHINENSIS CORTEX  REHMANNIAE RADIX |
| 2 | MOL000449 | stigmasterol | 43.83 | 0.76 | ANGELICAE SINENSIS RADIX  SCUTELLARIAE RADIX  PHELLODENDRI CHINENSIS CORTEX  REHMANNIAE RADIX PRAEPARATA |
| 3 | MOL001458 | coptisine | 30.67 | 0.86 | SCUTELLARIAE RADIX  COPTIDIS RHIZOMA  PHELLODENDRI CHINENSIS CORTEX |
| 4 | MOL000098 | quercetin | 46.43 | 0.28 | COPTIDIS RHIZOMA  PHELLODENDRI CHINENSIS CORTEX  ASTRAGALI RADIX |
| 5 | MOL000359 | sitosterol | 36.91 | 0.75 | REHMANNIAE RADIX PRAEPARATA  SCUTELLARIAE RADIX |
| 6 | MOL002897 | epiberberine | 43.09 | 0.78 | SCUTELLARIAE RADIX  COPTIDIS RHIZOMA |
| 7 | MOL001454 | berberine | 36.86 | 0.78 | COPTIDIS RHIZOMA  PHELLODENDRI CHINENSIS CORTEX |
| 8 | MOL013352 | obacunone | 43.29 | 0.77 | COPTIDIS RHIZOMA  PHELLODENDRI CHINENSIS CORTEX |
| 9 | MOL002894 | berberrubine | 35.74 | 0.73 | COPTIDIS RHIZOMA  PHELLODENDRI CHINENSIS CORTEX |
| 10 | MOL000622 | magnograndiolide | 63.71 | 0.19 | COPTIDIS RHIZOMA  PHELLODENDRI CHINENSIS CORTEX |
| 11 | MOL000762 | palmidin a | 35.36 | 0.65 | COPTIDIS RHIZOMA  PHELLODENDRI CHINENSIS CORTEX |
| 12 | MOL000785 | palmatine | 64.6 | 0.65 | COPTIDIS RHIZOMA  PHELLODENDRI CHINENSIS CORTEX |
| 13 | MOL002668 | worenine | 45.83 | 0.87 | COPTIDIS RHIZOMA  PHELLODENDRI CHINENSIS CORTEX |
| 14 | MOL000493 | campesterol | 37.58 | 0.71 | REHMANNIAE RADIX |
| 15 | MOL000519 | coniferin | 31.11 | 0.32 | REHMANNIAE RADIX |
| 16 | MOL000228 | (2r)-7-hydroxy-5-methoxy-2-phenylchroman-4-one | 55.23 | 0.2 | SCUTELLARIAE RADIX |
| 17 | MOL002913 | dihydrobaicalin_qt | 40.04 | 0.21 | SCUTELLARIAE RADIX |
| 18 | MOL000525 | norwogonin | 39.4 | 0.21 | SCUTELLARIAE RADIX |
| 19 | MOL002714 | baicalein | 33.52 | 0.21 | SCUTELLARIAE RADIX |
| 20 | MOL010415 | 11,13-eicosadienoic acid, methyl ester | 39.28 | 0.23 | SCUTELLARIAE RADIX |
| 21 | MOL000173 | wogonin | 30.68 | 0.23 | SCUTELLARIAE RADIX |
| 22 | MOL002937 | dihydrooroxylin | 66.06 | 0.23 | SCUTELLARIAE RADIX |
| 23 | MOL002928 | oroxylin a | 41.37 | 0.23 | SCUTELLARIAE RADIX |
| 24 | MOL001689 | acacetin | 34.97 | 0.24 | SCUTELLARIAE RADIX |
| 25 | MOL000073 | ent-epicatechin | 48.96 | 0.24 | SCUTELLARIAE RADIX |
| 26 | MOL002910 | carthamidin | 41.15 | 0.24 | SCUTELLARIAE RADIX |
| 27 | MOL002914 | eriodyctiol (flavanone) | 41.35 | 0.24 | SCUTELLARIAE RADIX |
| 28 | MOL002925 | 5,7,2',6'-tetrahydroxyflavone | 37.01 | 0.24 | SCUTELLARIAE RADIX |
| 29 | MOL008206 | moslosooflavone | 44.09 | 0.25 | SCUTELLARIAE RADIX |
| 30 | MOL012246 | 5,7,4'-trihydroxy-8-methoxyflavanone | 74.24 | 0.26 | SCUTELLARIAE RADIX |
| 31 | MOL002933 | 5,7,4'-trihydroxy-8-methoxyflavone | 36.56 | 0.27 | SCUTELLARIAE RADIX |
| 32 | MOL012245 | 5,7,4'-trihydroxy-6-methoxyflavanone | 36.63 | 0.27 | SCUTELLARIAE RADIX |
| 33 | MOL002932 | panicolin | 76.26 | 0.29 | SCUTELLARIAE RADIX |
| 34 | MOL002917 | 5,2',6'-trihydroxy-7,8-dimethoxyflavone | 45.05 | 0.33 | SCUTELLARIAE RADIX |
| 35 | MOL002915 | salvigenin | 49.07 | 0.33 | SCUTELLARIAE RADIX |
| 36 | MOL001490 | bis[(2s)-2-ethylhexyl] benzene-1,2-dicarboxylate | 43.59 | 0.35 | SCUTELLARIAE RADIX |
| 37 | MOL000552 | 5,2'-dihydroxy-6,7,8-trimethoxyflavone | 31.71 | 0.35 | SCUTELLARIAE RADIX |
| 38 | MOL012266 | rivularin | 37.94 | 0.37 | SCUTELLARIAE RADIX |
| 39 | MOL002879 | diop | 43.59 | 0.39 | SCUTELLARIAE RADIX |
| 40 | MOL002927 | skullcapflavone ii | 69.51 | 0.44 | SCUTELLARIAE RADIX |
| 41 | MOL002934 | neobaicalein | 104.34 | 0.44 | SCUTELLARIAE RADIX |
| 42 | MOL002909 | 5,7,2,5-tetrahydroxy-8,6-dimethoxyflavone | 33.82 | 0.45 | SCUTELLARIAE RADIX |
| 43 | MOL002926 | dihydrooroxylin a | 38.72 | 0.23 | SCUTELLARIAE RADIX |
| 44 | MOL002908 | 5,8,2'-trihydroxy-7-methoxyflavone | 37.01 | 0.27 | SCUTELLARIAE RADIX |
| 45 | MOL001506 | supraene | 33.55 | 0.42 | SCUTELLARIAE RADIX |
| 46 | MOL002903 | (r)-canadine | 55.37 | 0.77 | COPTIDIS RHIZOMA |
| 47 | MOL002904 | berlambine | 36.68 | 0.82 | COPTIDIS RHIZOMA |
| 48 | MOL002907 | corchoroside a_qt | 104.95 | 0.78 | COPTIDIS RHIZOMA |
| 49 | MOL008647 | moupinamide | 86.71 | 0.26 | COPTIDIS RHIZOMA |
| 50 | MOL002641 | phellavin_qt | 43.29 | 0.77 | PHELLODENDRI CHINENSIS CORTEX |
| 51 | MOL000787 | fumarine | 59.26 | 0.83 | PHELLODENDRI CHINENSIS CORTEX |
| 52 | MOL000790 | isocorypalmine | 35.77 | 0.59 | PHELLODENDRI CHINENSIS CORTEX |
| 53 | MOL001131 | phellamurin_qt | 56.6 | 0.39 | PHELLODENDRI CHINENSIS CORTEX |
| 54 | MOL001455 | (s)-canadine | 53.83 | 0.77 | PHELLODENDRI CHINENSIS CORTEX |
| 55 | MOL001771 | poriferast-5-en-3beta-ol | 36.91 | 0.75 | PHELLODENDRI CHINENSIS CORTEX |
| 56 | MOL005438 | campesterol | 37.58 | 0.71 | PHELLODENDRI CHINENSIS CORTEX |
| 57 | MOL006422 | thalifendine | 44.41 | 0.73 | PHELLODENDRI CHINENSIS CORTEX |
| 58 | MOL002636 | kihadalactone a | 34.21 | 0.82 | PHELLODENDRI CHINENSIS CORTEX |
| 59 | MOL002652 | delta7-dehydrosophoramine | 54.45 | 0.25 | PHELLODENDRI CHINENSIS CORTEX |
| 60 | MOL002656 | dihydroniloticin | 36.43 | 0.81 | PHELLODENDRI CHINENSIS CORTEX |
| 61 | MOL002643 | delta 7-stigmastenol | 37.42 | 0.75 | PHELLODENDRI CHINENSIS CORTEX |
| 62 | MOL002659 | kihadanin a | 31.6 | 0.7 | PHELLODENDRI CHINENSIS CORTEX |
| 63 | MOL002660 | niloticin | 41.41 | 0.82 | PHELLODENDRI CHINENSIS CORTEX |
| 64 | MOL002671 | candletoxin a | 31.81 | 0.69 | PHELLODENDRI CHINENSIS CORTEX |
| 65 | MOL002673 | hispidone | 36.18 | 0.83 | PHELLODENDRI CHINENSIS CORTEX |
| 66 | MOL006401 | melianone | 40.53 | 0.78 | PHELLODENDRI CHINENSIS CORTEX |
| 67 | MOL002644 | phellopterin | 40.19 | 0.28 | PHELLODENDRI CHINENSIS CORTEX |
| 68 | MOL002651 | dehydrotanshinone ii a | 43.76 | 0.4 | PHELLODENDRI CHINENSIS CORTEX |
| 69 | MOL002662 | rutaecarpine | 40.3 | 0.6 | PHELLODENDRI CHINENSIS CORTEX |
| 70 | MOL002663 | skimmianin | 40.14 | 0.2 | PHELLODENDRI CHINENSIS CORTEX |
| 71 | MOL002666 | chelerythrine | 34.18 | 0.78 | PHELLODENDRI CHINENSIS CORTEX |
| 72 | MOL002670 | cavidine | 35.64 | 0.81 | PHELLODENDRI CHINENSIS CORTEX |
| 73 | MOL002672 | hericenone h | 39 | 0.63 | PHELLODENDRI CHINENSIS CORTEX |
| 74 | MOL000211 | mairin | 55.38 | 0.78 | ASTRAGALI RADIX |
| 75 | MOL000392 | formononetin | 69.67 | 0.21 | ASTRAGALI RADIX |
| 76 | MOL000417 | calycosin | 47.75 | 0.24 | ASTRAGALI RADIX |
| 77 | MOL000422 | kaempferol | 41.88 | 0.24 | ASTRAGALI RADIX |
| 78 | MOL000433 | fa | 68.96 | 0.71 | ASTRAGALI RADIX |
| 79 | MOL000439 | isomucronulatol-7,2'-di-o-glucosiole | 49.28 | 0.62 | ASTRAGALI RADIX |
| 80 | MOL000442 | 1,7-dihydroxy-3,9-dimethoxy pterocarpene | 39.05 | 0.48 | ASTRAGALI RADIX |
| 81 | MOL000379 | 9,10-dimethoxypterocarpan-3-o-β-d-glucoside | 36.74 | 0.92 | ASTRAGALI RADIX |
| 82 | MOL000398 | isoflavanone | 109.99 | 0.3 | ASTRAGALI RADIX |
| 83 | MOL000438 | (3r)-3-(2-hydroxy-3,4-dimethoxyphenyl)chroman-7-ol | 67.67 | 0.26 | ASTRAGALI RADIX |
| 84 | MOL000239 | jaranol | 50.83 | 0.29 | ASTRAGALI RADIX |
| 85 | MOL000296 | hederagenin | 36.91 | 0.75 | ASTRAGALI RADIX |
| 86 | MOL000033 | (3s,8s,9s,10r,13r,14s,17r)-10,13-dimethyl-17-[(2r,5s)-5-propan-2-yloctan-2-yl]-2,3,4,7,8,9,11,12,14,15,16,17-dodecahydro-1h-cyclopenta[a]phenanthren-3-ol | 36.23 | 0.78 | ASTRAGALI RADIX |
| 87 | MOL000354 | isorhamnetin | 49.6 | 0.31 | ASTRAGALI RADIX |
| 88 | MOL000371 | 3,9-di-o-methylnissolin | 53.74 | 0.48 | ASTRAGALI RADIX |
| 89 | MOL000378 | 7-o-methylisomucronulatol | 74.69 | 0.3 | ASTRAGALI RADIX |
| 90 | MOL000380 | (6ar,11ar)-9,10-dimethoxy-6a,11a-dihydro-6h-benzofurano[3,2-c]chromen-3-ol | 64.26 | 0.42 | ASTRAGALI RADIX |
| 91 | MOL000387 | bifendate | 31.1 | 0.67 | ASTRAGALI RADIX |
